# An Exploratory Investigation of Heart Rate Variability in Response to Exercise Training and Detraining in Young and Middle-Aged Men

**DOI:** 10.3390/biology14070794

**Published:** 2025-06-30

**Authors:** Andres E. Carrillo, Petros C. Dinas, Paraskevi Gkiata, Alexa R. Ferri, Glen P. Kenny, Yiannis Koutedakis, Athanasios Z. Jamurtas, George S. Metsios, Andreas D. Flouris

**Affiliations:** 1 Department of Exercise Science, College of Health Sciences, Chatham University, Pittsburgh, PA 15232, USA; acarrillo@chatham.edu (A.E.C.); ferri.alexa@gmail.com (A.R.F.); 2 FAME Laboratory, Department of Physical Education and Sport Science, University of Thessaly, 42100 Trikala, Greece; petros.cd@gmail.com (P.C.D.); gkiata.vivi@gmail.com (P.G.); 3 Human and Environmental Physiology Research Unit, School of Human Kinetics, University of Ottawa, Ottawa, ON K1N 6N5, Canada; gkenny@uottawa.ca; 4 School of Physical Education, Sport Science, and Dietetics, University of Thessaly, 42100 Trikala, Greece; y.koutedakis@pe.uth.gr (Y.K.); ajamurt@pe.uth.gr (A.Z.J.); g.metsios@uth.gr (G.S.M.)

**Keywords:** aging, autonomic nervous system, cardiac autonomic modulation, physical activity, resistance training, aerobic, COVID-19

## Abstract

We examined heart rate (HR) and heart rate variability (HRV) in young and middle-aged men after a period of detraining that immediately followed the completion of an exercise training program. Eight young (27.8 ± 3.8 years) and ten middle-aged (41.9 ± 3.8 years) men were randomly assigned to complete an 8-week exercise training program that included either aerobic (young: *n* = 3; middle-aged: *n* = 2), resistance (young: *n* = 3; middle-aged: *n* = 3), or combined (aerobic/resistance) (young: *n* = 2; middle-aged: *n* = 5) exercise. Thereafter, participants ceased all planned exercise training activities during an 8-week detraining period. Resting HR and HRV were assessed at baseline, after exercise training, and after detraining. An analysis of mean differences between age groups at each time-point revealed a significantly higher standard deviation of normal RR intervals (SDNN), square root of the mean of squared differences between successive RR intervals (RMSSD), high-frequency (HF) band (0.15–0.40 Hz), and cardiac vagal index (CVI) in young participants when compared to middle-aged participants at baseline (*p* ≤ 0.019) and after detraining (*p* ≤ 0.045), but not after the 8-week exercise training intervention (*p* ≥ 0.057). Additionally, in middle-aged participants, we observed a significant negative association between the percent change in HRV indices (RMSSD, HF, and CVI) and systolic blood pressure in response to detraining (*p* < 0.05). In conclusion, young participants had higher levels of HRV indices at baseline, reflecting greater cardiac vagal modulation when compared to middle-aged participants. We showed that these age-related differences in HRV are diminished following exercise training but are reestablished following 8 weeks of detraining. Given that age-related attenuations in HRV may reflect changes in cardiovascular health, it is important to further investigate the relationships between HRV, exercise training and detraining, aging, and the risk of poor health outcomes.

## Simple Summary

Unexpected events such as injury or lockdowns during pandemics may lead to a sudden decrease in physical activity participation, resulting in detraining. Even short-term detraining has been shown to reverse gains induced by exercise training, potentially resulting in negative health consequences. Heart rate variability is a non-invasive measure of cardiac autonomic modulation, is associated with general health outcomes, and has been reported as sensitive to exercise training and detraining. It remains uncertain how heart rate variability changes in response to exercise training and detraining among individuals in different age groups. In the current study, we examined heart rate variability in young and middle-aged men in response to exercise training that was immediately followed by an equal period of detraining. Prior to exercise training, we observed an age difference—higher values in young participants—in heart rate variability that was less evident after exercise training. But after the detraining period, age differences in heart rate variability were similar to baseline levels. These results suggest that heart rate variability differences between age groups may fluctuate in response to exercise training and detraining. Changes in heart rate variability may reflect changes in cardiovascular health, emphasizing the importance of future work in studying the relationships between heart rate variability, physical activity, aging, and cardiovascular health outcomes.

## 1. Introduction

Heart rate variability (HRV) describes the cardiac autonomic nervous system-controlled fluctuation of time intervals between consecutive heart beats [1,2,3]. As such, HRV reflects the balance between parasympathetic and sympathetic nervous system activity, providing an important non-invasive indicator of physiological function and overall health. In normal resting conditions, an HRV profile that typically reflects vagal withdrawal and a shift in the balance of autonomic function towards sympathetic dominance (i.e., low HRV) has been associated with increased chronological age and a higher risk for the development of cardiovascular diseases [3,4,5,6]. For example, from the Framingham Heart Study, Singh et al. [6] found that low HRV was associated with an increased risk of developing hypertension, particularly in men—suggesting that autonomic dysfunction may be present in the early stages of hypertension. Conversely, high HRV at rest has been reported to reflect greater parasympathetic activity and has been associated with robust cardiovascular health and wellbeing [1].

Aerobic and/or resistance exercise training have been shown to attenuate age-related reductions in HRV by increasing cardiorespiratory fitness and cardiac vagal tone [7,8]. However, unexpected events such as injury or lockdowns during COVID-19 may disrupt physical activity participation [9,10,11,12], resulting in detraining and potentially placing individuals at an increased risk of metabolic and cardiovascular health deterioration. The consequences of suddenly imposed barriers to physical activity participation warrant careful attention, as emphasized in a recent review that reported an overall negative impact of lockdowns during COVID-19 on mobility, walking, and physical activity participation throughout Asia, Europe, and North America [13]. Even short-term disruptions in physical activity can result in health consequences that influence cardiovascular and autonomic function [14,15]. For example, cardiovascular autonomic adaptations in healthy young men observed after 12 weeks of exercise training were reversed following an 8-week detraining period [15]. It remains uncertain, however, how sudden exercise training cessation, resulting in a period of exercise detraining, may influence age-related attenuations in HRV, particularly among middle-aged populations. More importantly, the potential consequences of age-related disruptions in HRV due to exercise detraining on cardiovascular function are not fully understood.

To assess the impact of sudden exercise training cessation on age-related differences in cardiac autonomic modulation, we examined HRV in young and middle-aged men after an 8-week period of detraining that immediately followed the completion of an exercise training program. As a secondary aim, we assessed the relationship between changes in resting HRV and arterial blood pressure in response to detraining.

## 2. Materials and Methods

### 2.1. Participants

This study is part of a larger project that investigated the thermogenic activity of white adipose tissue in response to exercise training and detraining (clinical trial registration number: NCT04039685) [16]. Participants were recruited from the city of Trikala, Greece, through word of mouth, newspaper posts, and flyers. Non-smoking males between the ages of 18–59 years were eligible for participation. Individuals were excluded from study participation if they were current tobacco smokers, engaged in regular exercise training, diagnosed with a chronic disease, taking medication to treat a chronic condition, or if they had an orthopedic condition that would limit participation in an exercise training program.

The current study aimed to analyze a sub-sample of participants from the main project who completed heart rate variability (HRV) assessments throughout the study period [16]. Informed consent was obtained from 9 young (21–32 years) and 12 middle-aged (39–52 years) healthy men with low-to-moderate physical activity levels. Three volunteers (one young and two middle-aged) dropped out of the study due to time constraints. A total of 18 (8 young and 10 middle-aged) participants [height: 177.3 ± 6.1 cm, body mass: 87.5 ± 16.3 kg, body mass index (BMI): 27.6 ± 4.9 kg/m^2^, percent body fat: 29.2 ± 9.1%, and peak volume of oxygen consumption (VO_2_peak): 28.9 ± 8.8 mL/kg/min] successfully completed all study requirements. Physical activity levels were determined following completion of the International Physical Activity Questionnaire—a tool previously validated for the assessment of physical activity levels in healthy adults [17,18]. This study conformed to the standards set by the Declaration of Helsinki and was approved by the Ethics Committee at the University of Thessaly (protocol number: 698/13-3-2013).

### 2.2. Study Design

**Figure 1:**
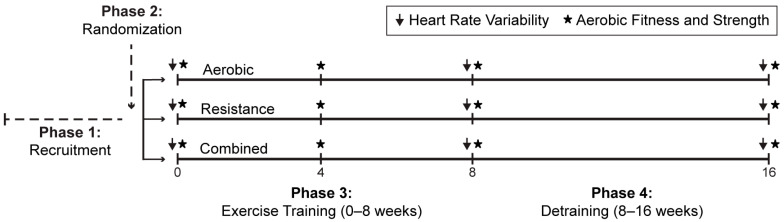
Study design. Arrow indicates assessment of heart rate variability. Asterisk indicates assessment of aerobic fitness and strength.

The current study employed a parallel-group design. The study period was divided into four phases (Figure 1): phase I—recruitment; phase II—randomization; phase III—exercise training intervention; and phase IV—detraining. Following recruitment (phase 1), participants were randomly assigned (random.org) (phase 2) to complete a supervised, 8-week moderate intensity exercise training program (phase 3) that included aerobic (young: 3; middle-aged: 2), resistance (young: 3; middle-aged: 3), or combined (aerobic and resistance) (young: 2; middle-aged: 5) exercise. An 8-week detraining period (phase 4) immediately followed the exercise training intervention. Outcome measurements (see Section 2.3) were assessed at baseline, after exercise training, and immediately following the detraining period.

### 2.3. Outcome Measurements

For data collection, participants visited the laboratory shortly after awakening, between 07:00 and 09:00, following a 12 h fast, and after refraining from exercise, alcohol, and passive smoking for 72 h prior to arrival. Anthropometry measurements included height and weight using a Seca device (Hamburg, Germany) and scale (KERN & Sohn GmbH, Version 5.3, Balingen, Germany), respectively. Percent body fat was measured using a bioelectrical impedance body composition analyzer (Fresenius Medical Care AG & Co. KGaA D-61346, Bad Homburg, Germany). Arterial blood pressure was measured after five minutes of seated rest using a standard aneroid sphygmomanometer (Medisave UK, Weymouth, Dorset, England). The mean value of two arterial blood pressure measurements (separated by two minutes) was used for statistical analysis.

Heart rate variability was measured using a Polar heart rate monitor (Polar RS800CX, Polar Electro Oy, Kempele, Finland) validated for HRV assessment [19]. During HRV measurements, participants remained in a resting, supine position for approximately 30 min in quiet, thermoneutral (22–24 °C and 40–60% relative humidity) conditions. Ten minutes of heart rate (HR) and time interval (RR) data were recorded, downloaded, and subsequently analyzed using the Kubios HRV analysis software V3.3. Five HRV indices that characterize most aspects of heart rhythm variability and complexity were extracted for statistical analysis [1,20]. Derived time-domain measures included the standard deviation of normal-to-normal intervals (SDNN) and the root mean square of successive RR interval differences (RMSSD)—both primarily influenced by the parasympathetic nervous system [20]. Frequency-domain measurements included the high-frequency (HF) band (0.15–0.40 Hz) (expressed as absolute power in ms^2^) that has been reported as a marker of vagal tone [1]. As a non-linear method, the cardiac vagal index was calculated [CVI = log_10_ (16 × SD1 × SD2)] to assess cardiac autonomic activity that is sensitive to vagal modulation [21]. Detrended fluctuation analysis alpha 1 (DFAα1) refers to the calculation of short-term fractal relationship properties of RR intervals and has been referred to as a clinically relevant marker that is sensitive to pathological processes [22].

Aerobic fitness and muscular strength as indexed by the peak volume of oxygen consumption (VO_2_peak) and 1-repetition maximum (RM, estimated for the leg and chest press), respectively, were assessed immediately following HRV measurements at each time-point and after 4 weeks of exercise training. Testing of VO_2_peak was conducted on a Monark Ergomedic 839E cycle ergometer (Vansbro, Sweden). After a 5 min warm-up period, participants cycled at 60 watts for 3 min, which was increased by an increment of 30 watts per minute until volitional exhaustion. A pedaling rate of 60 rpm was maintained throughout the test. The highest level of oxygen uptake measured from expired air using an automated gas analyzer (Vmax, CareFusion, Yorba Linda, CA, USA) was recorded as VO_2_peak. Following instruction on proper lifting technique, 1RMs for the leg press and chest press were estimated as previously described [16]. In brief, a suitable starting weight was selected such that each participant would achieve failure after completing <10 repetitions in no more than 3 sets. The weight (kg) lifted and the number of repetitions performed were used to estimate 1RM for the resistance training exercises.

### 2.4. Exercise Training and Detraining Protocol

Participants were randomly assigned to complete a supervised, 8-week moderate intensity exercise training program that included aerobic (young: 3; middle-aged: 2), resistance (young: 3; middle-aged: 3), or combined (aerobic and resistance) (young: 2; middle-aged: 5) exercise. Participants were wearing a Polar heart rate monitor and were supervised by an experienced investigator during each exercise session to ensure that the desired intensity and/or resistance were achieved. The exercise training programs were structured such that a combination of exercise frequency and cycling duration/number of sets would gradually increase, while the relative intensity (65% of VO_2_peak or estimated 1RM during) remained consistent between each group and throughout the 8-week training period. Resistance exercises included leg press, chest press, leg extension, leg curl, bicep curl, triceps extension, and latissimus dorsi pulldown. During the resistance exercise familiarization period (week 1), 1RM estimations were completed for all exercises to determine appropriate weights used during the resistance and combined exercise training programs. After 4 weeks of exercise training, VO_2_peak and 1RM estimations were completed again by participants in the aerobic/combined and resistance/combined training groups, respectively. If VO_2_peak or an estimated 1RM increased (compared to baseline), the cycling speed and/or weights were adjusted to maintain a workload equivalent to 65% of VO_2_peak or estimated 1RM during weeks 4–8. The exercise training programs are summarized in Table 1. Participants completed 94% of all scheduled exercise training sessions (aerobic: 93%; resistance: 97%; and combined: 92%). After completing the 8-week exercise training program, the detraining phase of the study was initiated. Participants no longer attended supervised exercise training sessions and were instructed to cease all planned exercise training activities/revert to their baseline physical activity levels for an additional period of 8 weeks.

### 2.5. Statistical Analysis

Participant characteristics, peak volume of oxygen consumption (VO_2_peak), and muscular strength (estimated 1RM for leg press and chest press) at baseline were compared between age groups (young and middle-aged) using independent-samples *t*-test. Changes in outcome variables after exercise training (i.e., value at 8 weeks after exercise training − value at baseline) and after detraining (i.e., value at 8 weeks after detraining − value at 8 weeks after exercise training) were compared between age groups using independent-samples *t*-test. Participant characteristics, VO_2_peak, and muscular strength data are presented as mean (standard deviation), and estimated mean differences between age groups are presented as mean (95% confidence interval).

Studentized residuals of HR and HRV (SDNN, RMSSD, HF, CVI, and DFAα1) data were assessed for normality using the Shapiro–Wilk test. Non-normal data were log-transformed prior to statistical analysis. Initial analyses were conducted to assess mean differences in HR and HRV indices across time [i.e., at baseline, at 8 weeks after exercise training, and at 8 weeks after exercise cessation (within-subjects factor)] and between exercise training modalities [i.e., aerobic, resistance, and combined (between-subjects factor)] using a two-way, mixed-model ANOVA. Mean differences in HR and HRV indices were compared between the young and middle-aged groups using a two-way, mixed-model ANOVA, both unadjusted and adjusted for baseline values. In unadjusted analyses, mean differences in HR and HRV indices were compared across time [i.e., at baseline, at 8 weeks after exercise training, and at 8 weeks after exercise cessation (within-subjects factor)] and between age groups [i.e., young and middle-aged (between-subjects factor)]. In adjusted analyses, baseline values (i.e., prior to exercise training) were included as a continuous covariate. Pairwise comparisons were interpreted using a Bonferroni adjustment when appropriate.

To estimate the magnitude of significant age-related differences in HR and HRV, effect size (*d*) was calculated and interpreted using Cohen’s criteria: 0.2–0.4 (small effect), 0.5–0.7 (medium effect), and ≥0.8 (large effect) [23]. Heart rate and HRV data are presented as mean (standard deviation), and estimated mean differences between age groups are presented as mean (95% confidence interval).

Pearson’s product-moment correlation was conducted to assess the relationship between the change in HRV indices and blood pressure in response to exercise detraining. Percent changes in HRV indices and blood pressure were calculated using the following equation: ((value at 8 weeks after detraining − value at 8 weeks after exercise training)/value at 8 weeks after exercise training) × 100. Data were analyzed using SPSS, version 29. Statistical significance was set at *p* < 0.05, and all tests were two-sided.

## 3. Results

### 3.1. Age-Related Differences in Participant Characteristics, Peak Volume of Oxygen Consumption, and Muscular Strength

Compared to the younger group, middle-aged participants were 14.2 years older (95% CI, −18.0 to −10.3, *t*(16) = −7.761, *p* < 0.001) and had 8.1% greater body fat (95% CI, −16.4 to 0.3, *t*(16) = −2.050, *p* = 0.057) but had a similar BMI (95% CI, −7.7 to 2.0, *t*(16) = −1.259, *p* = 0.226) and blood pressure [systolic: (95% CI, −5.1 to 16.0, *t*(16) = 1.101, *p* = 0.287), diastolic: (95% CI, −3.1 to 14.8, *t*(16) = 1.390, *p* = 0.184)] (Table 2).

The peak volume of oxygen consumption (95% CI, −4.6 to 13.0, *t*(16) = 1.009, *p* = 0.328), estimated 1RM leg press (95% CI, −58.2 to 21.8, *t*(11.176) = −0.998, *p* = 0.339), and estimated 1RM chest press (95% CI, −22.4 to 18.4, *t*(16) = −0.206, *p* = 0.840) were similar between young and middle-aged participants at baseline (Table 2). Systolic and diastolic blood pressure, peak volume of oxygen consumption, estimated 1RM leg press, and estimated 1RM chest press in response to exercise training and detraining are summarized in Table 2. Changes in blood pressure [systolic: (95% CI, −0.8 to 12.8, *t*(16) = 1.865, *p* = 0.081), diastolic: (95% CI, −6.4 to 6.8, *t*(16) = 0.064, *p* = 0.949)], VO_2_peak (95% CI, −5.3 to 6.2, *t*(15) = 0.161, *p* = 0.875), estimated 1RM leg press (95% CI, −49.4 to 6.7, *t*(8.759) = −1.729, *p* = 0.119), and estimated 1RM chest press (95% CI, −8.4 to 5.3, *t*(15) = −0.489, *p* = 0.632) from baseline to 8 weeks after exercise training (value at 8 weeks after exercise training − value at baseline) were similar between young and middle-aged participants. In response to detraining, changes (value at 8 weeks after detraining—value at 8 weeks after exercise training) in blood pressure [systolic: (95% CI, −11.1 to 0.7, *t*(16) = 1.879, *p* = 0.079), diastolic: (95% CI, −8.8 to 3.7, *t*(16) = 0.857, *p* = 0.404)], VO_2_peak (95% CI, −4.3 to 5.1, *t*(14) = 0.173, *p* = 0.865), estimated 1RM leg press (95% CI, −3.9 to 36.3, *t*(14) = 1.724, *p* = 0.107), and estimated 1RM chest press (95% CI, −5.5 to 8.1, *t*(14) = 0.418, *p* = 0.341) were similar between young and middle-aged participants.

### 3.2. Comparison of Heart Rate and Heart Rate Variability Between Exercise Modalities

No differences were observed in HR and HRV indices (SDNN, RMSSD, log HF, CVI, and DFAα1) within (main effect of time: *p* ≥ 0.323) and between exercise training groups (main effect of group: *p* ≥ 0.073). There were no statistically significant interactions between time and exercise training group (i.e., aerobic, resistance, or combined) on HR and HRV indices (*p*-values ranged from 0.574 to 0.995). Thus, HR and HRV responses to exercise training and detraining did not significantly differ between exercise modalities (i.e., aerobic, resistance, and combined).

### 3.3. Age-Related Differences in Heart Rate and Heart Rate Variability

In unadjusted analyses, there were no statistically significant interactions between time and age group on HR and HRV indices (*p* ≥ 0.657). A significant main effect of age group was observed in SDNN (*F*_1,16_ = 6.797, *p* = 0.019), RMSSD (*F*_1,16_ = 9.111, *p* = 0.008), log HF (*F*_1,16_ = 10.974, *p* = 0.004), and CVI (*F*_1,16_ = 8.784, *p* = 0.009). Further analysis of pairwise comparisons (Table 3) revealed significantly higher SDNN [mean difference: −15.7 (95% CI, −27.7 to −3.8, *p* = 0.013, *d* = 1.33)], RMSSD [mean difference: −19.1 (95% CI, −34.6 to −3.5, *p* = 0.019, *d* = 1.24)], log HF [mean difference: −0.5 (95% CI, −0.7 to −0.2, *p* = 0.005, *d* = 1.53)], and CVI [mean difference: −0.4 (95% CI, −0.6 to −0.1, *p* = 0.007, *d* = 1.47)] among young participants when compared to middle-aged participants at baseline (Table 3). After 8 weeks of exercise training, however, SDNN (*p* = 0.109), RMSSD (*p* = 0.100), log HF (*p* = 0.057), and CVI (*p* = 0.062) were no longer significantly different between age groups (young and middle-aged). Age-related differences in HRV indices returned after 8 weeks of detraining. Specifically, SDNN [mean difference: −16.7 (95% CI, −32.9 to −0.5, *p* = 0.045, *d* = 1.03)], RMSSD [mean difference: −23.2 (95% CI, −38.5 to −8.0, *p* = 0.005, *d* = 1.53)], log HF [mean difference: −0.5 (95% CI, −0.8 to −0.2, *p* = 0.002, *r* = 1.78)], and CVI [mean difference: −0.4 (95% CI, −0.7 to −0.1, *p* = 0.012, *d* = 1.35)] were significantly higher among young participants when compared to middle-aged participants following the 8-week detraining period (Table 3). Except for RMSSD, mean differences in HRV indices (SDNN, log HF, and CVI) between age groups were no longer statistically significant following adjustment for baseline values (*p* > 0.05). In adjusted analyses, RMSSD was similar between age groups after exercise training (*p* = 0.763) but was significantly higher in young participants when compared to middle-aged participants after the 8-week detraining period [mean difference: −18.6 (95% CI, −37.0 to −0.3, *p* = 0.047)] (Table 3). In adjusted analyses, there were no statistically significant interactions between time and age group in HR and HRV indices (*p* ≥ 0.08).

**Figure 2:**
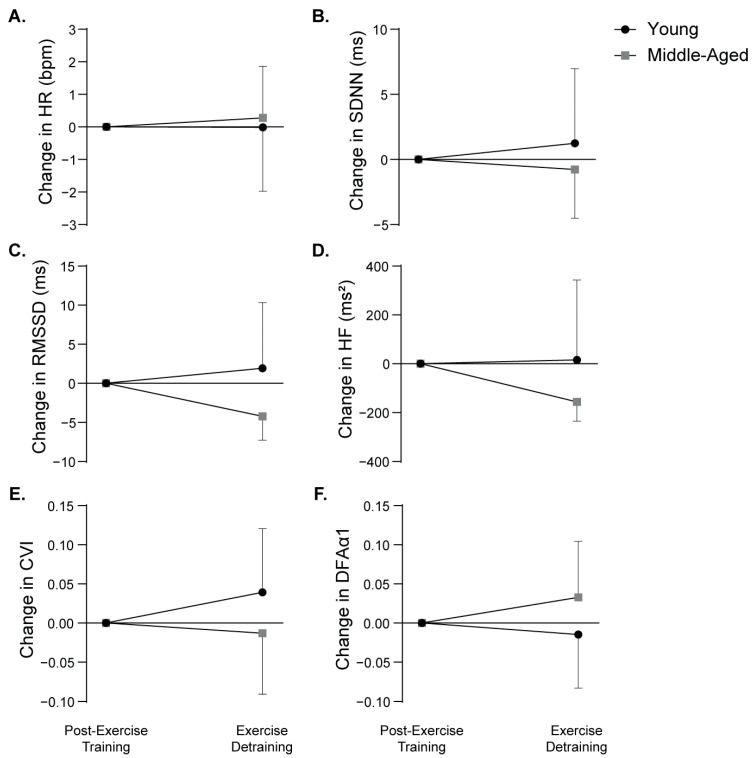
Change in heart rate (HR) (**A**) and heart rate variability (HRV) indices following 8 weeks of detraining in young (*n* = 8) and middle-aged (*n* = 10) participants. Heart rate variability indices include the standard deviation of normal RR intervals (SDNN) (**B**), the square root of the mean of squared differences between successive RR intervals (RMSSD) (**C**), high-frequency (HF) band (0.15–0.40 Hz) (**D**), cardiac vagal index (CVI) (**E**), and detrended fluctuation analysis alpha 1 (DFAα1) (**F**). Changes in HR and HRV indices were calculated using the following equation: value at 8 weeks after detraining—value at 8 weeks after exercise training. Data are presented as mean (SEM). No significant differences were observed between age groups (*p* ≥ 0.468).

In both unadjusted and adjusted analyses, HR and DFAα1 between age groups were similar (*p* > 0.05). In both unadjusted and adjusted analyses, there were no significant main effects of time in HR and HRV indices within each age group (*p* > 0.05). Finally, the changes in HRV indices in response to detraining (value at 8 weeks after detraining − value at 8 weeks after exercise training) were similar between age groups (*p* ≥ 0.468) (Figure 2).

### 3.4. Associations Between Percent Change in Heart Rate Variability Indices and Arterial Blood Pressure in Response to Exercise Detraining

Results from Pearson’s product-moment correlation revealed no statistically significant associations between the percent change in HRV indices (i.e., SDNN, RMSSD, LF, HF, CVI, DFAα1) and blood pressure (i.e., systolic and diastolic), in response to exercise detraining, when young and middle-aged participants were combined. In young participants, the percent change in diastolic blood pressure was negatively associated with the percent change in DFAα1 [r(6) = −0.772, *p* = 0.025]. Among middle-aged participants, the percent change in systolic blood pressure was negatively associated with the percent change in RMSSD [r(8) = −0.740, *p* = 0.014], HF [r(8) = −0.848, *p* = 0.002], and CVI [r(8) = −0.668, *p* = 0.035], whereas the percent change in diastolic blood pressure was positively associated with the percent change in heart rate [r(8) = 0.643, *p* = 0.045]. No other statistically significant correlations between the percent change in HRV indices and blood pressure, in response to exercise detraining, were observed in young or middle-aged participants (*p* > 0.05).

## 4. Discussion

We found that age-related differences in HRV are diminished following exercise training but are reestablished following 8 weeks of detraining. Prior to exercise training, SDNN, RMSSD, log HF, and CVI (all with a large effect size) were significantly higher in young participants when compared to middle-aged participants. These results are consistent with previous findings and reflect an age-related decline in cardiac vagal activity beginning in some individuals by the age of 30 years [3,24]. Although some controversy exists [25,26], an HRV profile that reflects low cardiac vagal tone (e.g., low RMSSD), occurring due to increased chronological age or other factors (e.g., obesity or physical inactivity), seems to occur in parallel with, and may reflect, a gradual decline in overall cardiovascular health [27,28], emphasizing the importance of implementing lifestyle habits that increase HRV. For example, exercise training and high cardiorespiratory fitness are consistently shown to be associated with favorable changes in HRV, particularly among older adults [9,29]. From the CARDIA study [29], a cohort of 2316 participants was assessed for HRV, cardiorespiratory fitness, and anthropometry, at baseline and after 20 years. The participants with lower cardiorespiratory fitness and higher measures of adiposity were more likely to have low HRV, although cardiorespiratory fitness was found to be the stronger determinant. There is some evidence supporting both aerobic and resistance exercise training as effective exercise modalities for improving HRV among individuals in different age groups [7,30,31]. Although evidence generally supports aerobic exercise training as the superior mode for improving HRV [7,31], the potential for any exercise training program to positively alter resting HRV depends on multiple factors such as training volume, chronological age, and cardiorespiratory fitness level. For example, resistance training has been shown to be less effective at influencing HRV in young, healthy adults [32], whereas individuals with autonomic dysfunction or chronic disease tend to respond more favorably [33,34]. In the current study, however, the type of exercise performed did not significantly influence HRV outcomes in response to exercise training.

Age-related differences in HRV indices observed at baseline were no longer significant after 8 weeks of aerobic and/or resistance training. This suggests that 8 weeks of exercise training may have contributed, at least in part, to reducing the severity of age-related attenuations in HRV observed in middle-aged men. The age-related HRV differences, however, in SDNN, RMSSD, HF, and CVI (all with a large effect size) drifted towards baseline levels after an 8-week period of detraining. These changes, observed after detraining, were likely due to a greater decline in HRV among the middle-aged compared to young men. For example, the young and middle-aged men experienced an average change of 15.6 (1.4%) ms^2^ and −156.3 (−28.4%) ms^2^, respectively, in HF from the onset of exercise cessation to the end of the 8-week detraining period. Similar to the unadjusted analyses, when HRV metrics were adjusted for baseline values, age-related differences in RMSSD were insignificant after exercise training but were significant after the 8-week detraining period. This suggests that HRV among the middle-aged men may have been more sensitive to the sudden cessation of exercise, whereas the younger men appeared to be more robust. However, it is important to note that age-related differences in SDNN, log HF, and CVI were no longer statistically significant after adjusting for baseline values.

The apparent return of age-related differences in HRV after detraining reflects a possible recurrence of reduced parasympathetic modulation that may reflect a shift towards impaired metabolic and cardiovascular function among the middle-aged participants. Other studies have shown that improvements in HRV observed after a period of exercise training diminish following 4 and 8 weeks of detraining in older and young adults, respectively [15,35]. Heffernan et al. [32] reported no change in linear HRV metrics but did observe an increase in sample entropy—a measure of cardiac complexity associated with cardiovascular health—in young men following 6 weeks of resistance training that returned to baseline levels after 4 weeks of detraining. Our results are partially aligned with those by Heffernan et al. [32] given that we did not observe a change in HRV indices within each age group following exercise training and detraining, which was likely due to a low sample size and marked heterogeneity of response. We did, however, observe notable attenuations of age-related differences in HRV following exercise training, which regressed towards baseline levels following the detraining period.

We observed a significant negative association between the percent change in HRV indices and systolic blood pressure in response to detraining. Specifically, a greater percent decrease in HRV indices reflective of cardiac vagal modulation (i.e., RMSSD, HF, and CVI) was associated with a greater percent increase in systolic blood pressure. These relationships were more evident within the middle-aged group and support the notion that age-related changes in HRV following an 8-week period of detraining may reflect a gradual decline in cardiovascular health. Our findings support previous work suggesting that disruption in cardiac autonomic modulation is associated with high blood pressure [6,36]. For example, a study conducted in 146 individuals with and without hypertension (~53 years of age) found that HRV indices such as RMSSD and HF were lower among hypertensives, suggesting a disturbance in cardiac autonomic balance in essential hypertension [36]. Given that age-related attenuations in HRV may reflect a decline in overall cardiovascular health, our findings emphasize the need for further investigation of relationships between HRV, exercise training and detraining, aging, and the risk of poor health outcomes.

It is reasonable to assume that the present results may have been influenced by methodological limitations. It is important, for example, to emphasize our low sample size, which may, at least in part, indicate why no statistically significant time by age group interactions were observed despite the reported significant main effects of age group in HRV indices. Given that the current work was an exploratory investigation, an a priori sample size calculation was not completed and should be considered for future work. Another limitation is that participants completed one of three different exercise training programs. Yet, the relative intensity remained consistent, and HRV responses were similar between exercise training modalities. An assessment of chronotype, which has been shown to influence HRV in relation to tasks performed at a certain time of the day, was not included in the current study [37,38,39]. It is also important to emphasize that other factors, such as psychological stress, which may be experienced during situations resulting in sudden exercise cessation, such as in response to the COVID-19 lockdowns, were not considered for this study. Finally, we limited our interpretation of HRV to only five indices that have been consistently reported as sensitive to cardiac vagal modulation and/or clinically relevant [1,20,21,22]. Indices that have been suggested to reflect sympathetic activity or sympathovagal modulation were excluded from the current analysis due to inconsistencies reported in the literature [20]. We acknowledge that future work may benefit from the inclusion of additional HRV indices.

## 5. Conclusions

As expected, young participants had higher levels of HRV indices at baseline, reflecting greater cardiac vagal modulation when compared to middle-aged participants. After 8 weeks of exercise training, HRV indices were similar between age groups, supporting the notion that exercise is beneficial in improving cardiac autonomic regulation, contributing to a healthy HRV profile. Eight weeks of detraining, however, reestablished age-related differences in HRV indices. Therefore, public health measures should address physical activity challenges and barriers associated with situations resulting in abrupt exercise cessation or detraining, such as home confinement, with alternative strategies such as at-home measures, to promote and maintain active lifestyles, societal wellness, and health equity.

## Figures and Tables

**Table 1:** Exercise training programs.

Exercise Modality	Week			
	1	2	3	4–8
Aerobic	30 min of cycling at 65% VO_2_peak 3 days/week	45 min of cycling at 65% VO_2_peak 3 days/week	45 min of cycling at 65% VO_2_peak 5 days/week	60 min of cycling at 65% VO_2_peak 5 days/week
Resistance	* Resistance training familiarization session 4 days/week	2 sets of 8–10 reps at 65% of 1RM 4 days/week	3 sets of 8–10 reps at 65% of 1RM 4 days/week	4 sets of 8–10 reps at 65% of 1RM 4 days/week
Combined	15 min of cycling at 65% VO_2_peak and * resistance training familiarization session 3 days/week	23 min of cycling at 65% VO_2_peak and 1 set of 8–10 reps at 65% of 1RM 4 days/week	23 min of cycling at 65% VO_2_peak and 2 sets of 8–10 reps at 65% of 1RM 4 days/week	30 min of cycling at 65% VO_2_peak and 2 sets of 8–10 reps at 65% of 1RM 5 days/week

Resistance exercises included leg press, chest press, leg extension, leg curl, bicep curl, triceps extension, and latissimus dorsi pulldown. Peak volume of oxygen consumption (VO_2_peak); estimated one-repetition maximum (1RM). * Resistance training familiarization session included an introduction to the equipment and the completion of 1RM estimations for all resistance exercises.

**Table 2:** Participant characteristics, cardiorespiratory fitness, and muscular strength.

Variable	Young (*n* = 8)			Middle-Aged (*n* = 10)		
	Baseline	Exercise Training	Detraining	Baseline	Exercise Training	Detraining
Age (years)	27.8 (3.8)	--	--	41.9 (3.8) *	--	--
BMI (kg/m^2^)	26.0 (4.9)	26.3 (5.0)	26.1 (4.9)	28.9 (4.7)	29.2 (4.7)	29.2 (5.1)
Percent BF (%)	24.7 (8.8)	24.9 (8.1)	25.4 (8.6)	32.8 (7.9)	32.3 (8.6)	32.3 (7.7)
Systolic BP (mmHg)	120.8 (9.8)	112.8 (10.0)	116.9 (11.1)	126.2 (10.9)	124.2 (13.0)	123.1 (12.7)
Diastolic BP (mmHg)	81.6 (7.6)	79.1 (4.2)	82.3 (8.7)	87.5 (9.8)	85.2 (12.2)	85.8 (10.6)
VO_2_peak (mL/kg/min)	31.2 (8.5)	34.1 (7.8)	32.4 (7.3)	27.0 (9.0)	30.8 (8.3)	29.2 (7.4)
1RM Leg Press (kg)	67.0 (17.2)	69.8 (14.1)	67.1 (13.4)	85.1 (54.3)	112.2 (87.2)	95.9 (69.5)
1RM Chest Press (kg)	56.0 (19.7)	58.9 (22.4)	56.1 (19.9)	58.0 (20.8)	62.2 (19.1)	57.6 (21.6)

Presented values were measured at baseline, after 8 weeks of exercise training, and at 8 weeks after exercise cessation (detraining). Values are presented as mean (SD). * indicates a significant difference from young participants at baseline (*p* < 0.05). Body mass index (BMI), body fat (BF), blood pressure (BP), peak volume of oxygen consumption (VO_2_peak), and estimated one-repetition maximum (1RM).

**Table 3:** Heart rate and heart rate variability responses to exercise training and detraining in young and middle-aged men.

Variable	Mean (SD)		Unadjusted Difference		Adjusted Difference	
	Young (*n* = 8)	Middle-Aged (*n* = 10)	Mean [95% CI]	*p*-Value	Mean [95% CI]	*p*-Value
Heart Rate (bpm)						
Baseline	62.2 (5.4)	62.2 (7.2)	0.0 [−6.5, 6.6]	0.996	–	–
Exercise Training	60.6 (4.3)	61.5 (8.6)	0.8 [−6.3, 8.0]	0.805	0.8 [−5.1, 6.80]	0.771
Detraining	60.6 (4.9)	61.7 (7.0)	1.1 [−5.1, 7.3]	0.709	1.1 [−3.0, 5.2]	0.577
SDNN (ms)						
Baseline	50.0 (11.7)	34.3 (12.0)	−15.7 [−27.7, −3.9]	0.013 *	–	–
Exercise Training	54.3 (15.8)	39.6 (19.8)	−14.6 [−32.9, 3.6]	0.109	−6.2 [−27.8, 15.5]	0.554
Detraining	55.6 (13.9)	38.9 (17.7)	−16.7 [−32.9, −0.5]	0.045 *	−7.2 [−25.7, 11.3]	0.417
RMSSD (ms)						
Baseline	51.7 (19.3)	32.6 (11.6)	−19.1 [−34.6, −3.5]	0.019 *	–	–
Exercise Training	56.6 (30.6)	39.5 (20.8)	−17.1 [−37.9, 3.7]	0.100	−3.1 [−24.7, 18.5]	0.763
Detraining	58.5 (15.1)	35.3 (15.2)	−23.2 [−38.5, −8.0]	0.005 *	−18.6 [−37.0, −0.3]	0.047 *
Log HF						
Baseline	2.9 (0.3)	2.5 (0.3)	−0.5 [−0.8, −0.2]	0.005 *	–	–
Exercise Training	2.9 (0.3)	2.6 (0.5)	−0.4 [−0.8, 0.0]	0.057	0.0 [−0.5, 0.4]	0.841
Detraining	3.0 (0.2)	2.5 (0.3)	−0.5 [−0.8, −0.2]	0.002 *	−0.3 [−0.6, 0.0]	0.084
CVI						
Baseline	4.5 (0.3)	4.1 (0.3)	−0.4 [−0.6, −0.1]	0.007 *	–	–
Exercise Training	4.6 (0.2)	4.2 (0.5)	−0.4 [−0.8, 0.0]	0.062	−0.1 [−0.6, 0.4]	0.657
Detraining	4.6 (0.2)	4.2 (0.4)	−0.4 [−0.7, −0.1]	0.012 *	−0.2 [−0.5, 0.2]	0.319
DFAα1						
Baseline	1.0 (0.2)	1.1 (0.3)	0.1 [−0.2, 0.3]	0.581	–	–
Exercise Training	1.0 (0.3)	1.1 (0.2)	0.0 [−0.2, 0.3]	0.748	0.0 [−0.2, 0.2]	0.897
Detraining	1.0 (0.1)	1.1 (0.3)	0.1 [−0.2, 0.3]	0.477	0.0 [−0.2, 0.2]	0.648

Heart rate (HR) and heart rate variability (HRV) raw data are presented as mean (standard deviation), and mean differences between age groups (middle-aged−young) are presented as mean (95% confidence interval). Mean differences between age groups were compared using a two-way, mixed-model ANOVA, both unadjusted and adjusted for baseline values. In unadjusted analyses, mean differences in HR and HRV indices were compared across time (at baseline, at 8 weeks after exercise training, and at 8 weeks after exercise detraining) and between age groups (young and middle-aged). In adjusted analyses, baseline values were included as a continuous covariate. * indicates a significant mean difference between age groups. Standard deviation of normal RR intervals (SDNN), square root of the mean of squared differences between successive RR intervals (RMSSD), high-frequency (HF) band (0.15–0.40 Hz), cardiac vagal index (CVI), and detrended fluctuation analysis alpha 1 (DFAα1).

## Data Availability

Data are available from the corresponding author upon reasonable request.

## Abbreviations

The following abbreviations are used in this manuscript:

1RM	One-repetition maximum
BMI	Body mass index
COVID-19	Coronavirus disease 2019
CVI	Cardiac vagal index
DFAα1	Detrended fluctuation analysis alpha 1
HF	High frequency
HR	Heart rate
HRV	Heart rate variability
RMSSD	Square root of the mean of squared differences between successive RR intervals
RR	Time interval between heart beats
SDNN	Standard deviation of normal RR intervals
VO_2_peak	Peak volume of oxygen consumption
